# Transcriptome analysis of atemoya pericarp elucidates the role of polysaccharide metabolism in fruit ripening and cracking after harvest

**DOI:** 10.1186/s12870-019-1756-4

**Published:** 2019-05-27

**Authors:** Jingjing Chen, Yajie Duan, Yulin Hu, Weiming Li, Dequan Sun, Huigang Hu, Jianghui Xie

**Affiliations:** 1 Key Laboratory of Tropical Fruit Biology, Ministry of Agriculture, South Subtropical Crops Research Institute, Chinese Academy of Tropical Agricultural Sciences, Zhanjiang, 524091 China; 2 National Field Genebank for Tropical Fruit (Zhanjiang), South Subtropical Crops Research Institute, Chinese Academy of Tropical Agricultural Sciences, Zhanjiang, 524091 China

## Abstract

**Background:**

Mature fruit cracking during the normal season in African Pride (AP) atemoya is a major problem in postharvest storage. Our current understanding of the molecular mechanism underlying fruit cracking is limited. The aim of this study was to unravel the role starch degradation and cell wall polysaccharide metabolism in fruit ripening and cracking after harvest through transcriptome analysis.

**Results:**

Transcriptome analysis of AP atemoya pericarp from cracking fruits of ethylene treatments and controls was performed. KEGG pathway analysis revealed that the starch and sucrose metabolism pathway was significantly enriched, and approximately 39 DEGs could be functionally annotated, which included starch, cellulose, pectin, and other sugar metabolism-related genes. Starch, protopectin, and soluble pectin contents among the different cracking stages after ethylene treatment and the controls were monitored. The results revealed that ethylene accelerated starch degradation, inhibited protopectin synthesis, and enhanced the soluble pectin content, compared to the control, which coincides with the phenotype of ethylene-induced fruit cracking. Key genes implicated in the starch, pectin, and cellulose degradation were further investigated using RT-qPCR analysis. The results revealed that alpha-amylase 1 (*AMY1*), alpha-amylase 3 (*AMY3*), beta-amylase 1 (*BAM1*), beta-amylase 3 (*BAM3*), beta-amylase 9 (*BAM9)*, pullulanase (*PUL)*, and glycogen debranching enzyme (*glgX*), were the major genes involved in starch degradation. *AMY1*, *BAM3*, *BAM9*, *PUL*, and *glgX* all were upregulated and had higher expression levels with ethylene treatment compared to the controls, suggesting that ethylene treatment may be responsible for accelerating starch degradation. The expression profile of alpha-1,4-galacturonosyltransferase (*GAUT*) and granule-bound starch synthase (*GBSS*) coincided with protopectin content changes and could involve protopectin synthesis. Pectinesterase (*PE*), polygalacturonase (*PG*), and pectate lyase (*PEL*) all involved in pectin degradation; *PE* was significantly upregulated by ethylene and was the key enzyme implicated pectin degradation.

**Conclusion:**

Both KEGG pathway enrichment analysis of DEGs and material content analysis confirmed that starch decomposition into soluble sugars and cell wall polysaccharides metabolism are closely related to the ripening and cracking of AP atemoya. A link between gene up- or downregulation during different cracking stages of atemoya fruits and how their expression affects starch and pectin contents were established by RT-qPCR analysis.

**Electronic supplementary material:**

The online version of this article (10.1186/s12870-019-1756-4) contains supplementary material, which is available to authorized users.

## Background

African Pride (AP) belongs to atemoya (*Annona cherimola* Mill × *A. squamosa* L.), a semi-deciduous, exotic subtropical fruit that is consumed in various countries [1]. It readily softens and cracks, thus reducing marketable yield and quality, and in turn promotes disease [2]. Fruit cracking during atemoya ripening initially develops around the peduncle and then radiates outward from the fruit base. It can occur before and after harvest, and the amount varies with cultivar. A wide range of stimuli that varies among species triggers fruit cracking. Correlations have been observed between susceptibility of fruit cracking and specific fruit traits, such as shape [3], size [4], firmness [5], stomata in fruit skin [6], cuticular properties [7], osmotic concentration, and other factors [8]. Fruit cracking in AP atemoya more commonly occurs during postharvest storage. Some measures have been adopted to delay cracking, including low temperature storage, 1-methylcyclopropene (1-MCP) treatment [9], chitosan and citric acid treatment [10], and salicylic acid treatment [11]. However, these could not effectively prevent fruit cracking, and our current understanding of the molecular mechanism underlying fruit cracking is limited.

Atemoya is a fruit with typical climacteric maturation, and studies have shown that fruit cracking starts at the respiratory peak and as ethylene production increases [2, 12–14]. Generally, atemoya softening is accompanied by cracking. Sugar metabolism is the most important material transformation process in atemoya fruit ripening, including starch degradation, transformation, and cell polysaccharides metabolism. It has been reported that fruit softening is typically accompanied by the depolymerization and solubilization of various classes of cell wall polysaccharides, such as pectins and hemicelluloses, and by elevated levels of the expression of genes, proteins, and enzyme activities associated with cell wall degradation [15, 16]. However, our current understanding of the molecular mechanism underlying fruit cracking is limited.

Only a few studies on the elucidation of the molecular mechanism of atemoya cracking have been conducted to date. Several cell wall-related genes, cell wall relaxation factors, and cuticle-related genes have been identified, including polygalacturonase (*PG*), pectinesterase (*PE*), β-galactosidase (*β*-G*al*), expansins (*EXP*), xyloglucan endotransglycosylase (*XET*), AP2/EREBP-type transcription factor (*PaWINB*), wax synthase (*WS*), and β-ketoacyl-CoA synthase (*PaKCS6*) [8, 17–23], are associated with fruit softening and cracking. Whole genome sequencing of atemoya has not been performed to date, and only a few genetic resources are currently available. The objective of this study was to identify cracking-related metabolic pathways and genes in atemoya fruits after ethylene treatment and in controls during postharvest through transcriptome sequencing and to establish a solid foundation for future molecular studies using high-throughput sequencing and expression data analysis.

## Results

### Appearance, firmness, and TSS changes in atemoya fruits after ethylene treatment and in controls

**Fig. 1:**
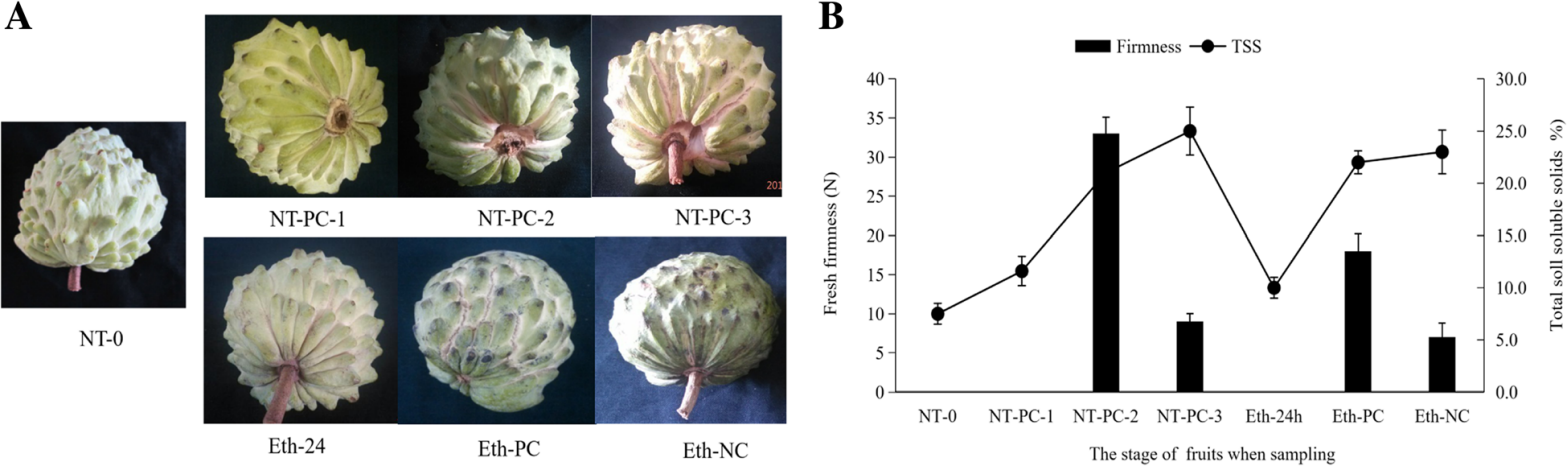
Appearance, firmness, and TSS content of atemoya fruits at postharvest. **a** Atemoya fruits during postharvest storage with or without ethylene treatment. Samples corresponding to various cracking degrees designated as NT-0, NT-PC-1, NT-PC-2, and NT-PC-3 for normal treatment and Eth-24 h, Eth-PC, and Eth-NC for the ethylene treatment were selected for transcriptome sequencing. **b** Firmness values and TSS contents after ethylene and no treatments. Vertical bars represent the standard error of three biological replicates

AP summer fruits are generally ready to eat about 3 or 4 days after fruit harvest (80% ripening). When the skin color changes from dark green to light green [14, 24], the surface of the fruit is cream in color between segments, and the surface of the separate fruit carpels becomes smooth [2], the fruits are harvested. However, in addition to the above two conditions, the definition of 80% ripening also includes a 10% soluble solids content [24]. Under room temperature and in the summer in Zhanjiang City, China, fruits started to separate between the peduncle and central axis 2 days after harvest as shown as NT-PC-1 in Fig. 1a. We used penetration tests to evaluate atemoya softening and found that the firmness value of fruits was too high to determine, but TSS levels at day 2 (11.6%) were higher than those on day 0/NT-0 (9.5%). On day 3, cracking radiated out from the fruit base, as shown as NT-PC-2 in Fig. 1a, and the fruits started to soften, allowing us to measure firmness. The TSS levels continued to increase to 21%. On day 4, cracking was deeper, radiating from the peduncle to the fruit top in some fruits, thereby completely exposing the peduncle as shown as NT-PC-3 in Fig. 1a. The firmness of the fruits rapidly decreased, and the highest TSS values (25%) were observed.

After 24 h of ethylene treatment (Eth-24 h), no difference in appearance between atemoya fruits on day 0/NT-0 was observed, although TSS content was slightly higher than that on day 0/NT-0. On day 2, the peel started to show signs of cracking, and regions around the peel had softened. On the third day, the fruits showed more extensive and deeper cracking, including the peduncle as shown as Eth-PC in Fig. 1a. Fruit firmness rapidly decreased and the highest TSS values were observed (Fig. 1b). However, some fruits subjected to ethylene treatment did not show signs of cracking, but exhibited similar changes in firmness and TSS content as that in cracking fruits shown as Eth-NC in Fig. 1.

### Transcriptome sequencing, assembly, and annotation

**Fig. 2:**
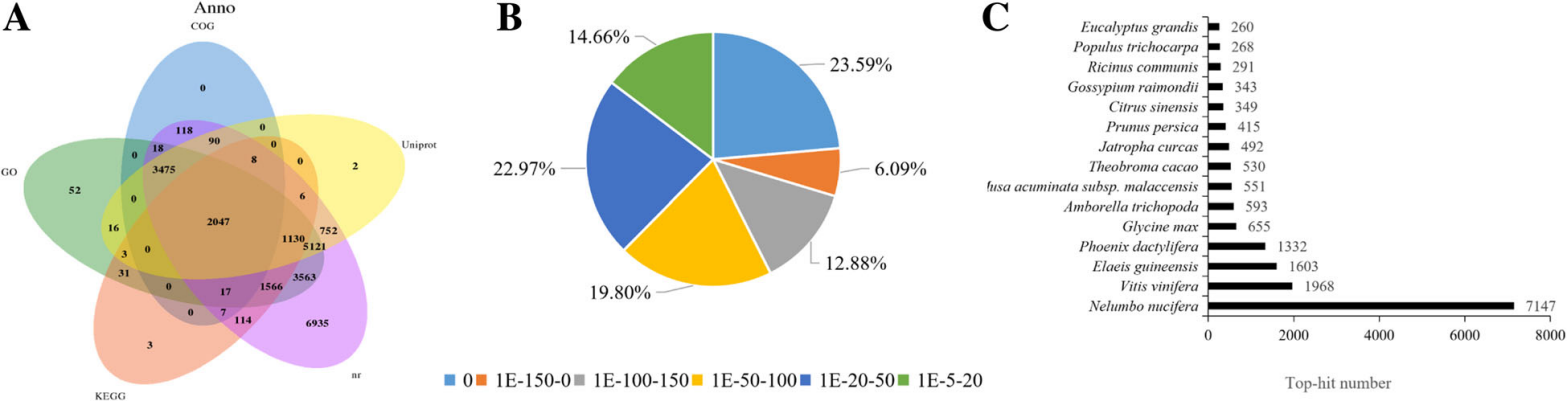
Characteristics of homology search of atemoya unigenes. **a** Venn diagram of number of unigenes annotated by BLASTx with a cut-off E-value 1e− 05 against protein databases. Numbers in the circles indicate the number of unigenes annotated by single or multiple databases. **b** E-value distribution of the top BLASTx hits against the Nr database. **c** Number of unigenes matching the 25 top species using BLASTx in the Nr database

To obtain a reference transcriptome for cracking atemoya fruits, a RNA-Seq library was constructed using RNAs from the pericarp, and seven libraries were generated from untreated fruits at room temperature (NT-0, NT-PC-1, NT-PC-2, and NT-PC-3) and ethylene-treated fruits (Eth-24 h, Eth-PC, and Eth-NC) at different cracking phases. These libraries were sequenced using the Illumina Hiseq™ 2500 platform. Table 1 shows that we generated a total of 38 Gb of nucleotides with a Q30 percentage of 95%, and the percentage of unassigned “N” bases was nearly 0. The distribution of reads in the seven libraries was 50.9 million (13.34%) for the control (NT-0), 57.1 million (14.97%) for NT-PC-1, 49.4 million (12.95%) for NT-PC-2, 50.0 (13.10%) for NT-PC-3, and 69.58 million (14.97%) for Eth-24 h, 47.4 (18.23%) for Eth-PC, and 38.2 (12.42%) for Eth-NC. After cleaning and performing quality checks, the Trinity package was used to performed de novo assembly of 64,069 unigenes, with a mean size of 670 bp. The size distributions of these unigenes are shown in Additional file 1. After searching for homologous sequences using BLASTX against Nr, UniProt, GO, KEGG, and COG databases, a total of 39.14% (25,074/64,069) unigenes could be assigned to a homolog in all five databases (Fig. 2a). About 38.97% (24,967/64,069) of the unigenes could be annotated by BLASTx (E-value <1e-5) using the NCBI Nr database. Based on the E-value distribution, we found that approximately 42.56% of the unigenes showed very strong homology (E-value <1e-100) to available plant sequences (Fig. 2b). Figure 2c shows that approximately 12,705 unigenes were annotated to five top-hit species, including *Nelumbo nucifera*, *Vitis vinifera*, *Elaeis guineensis*, and *Glycine max*.

**Table 1:** Throughput and quality of RNA-seq of the reference library and the DGE libraries

Libraries	Clean Reads Number	Clean Reads Rate (%)	Clean Bases Number	Ns Reads Number	Ns Reads Rate (%)	Clean Q30 Bases Rate (%)
NT-0	50,896,590	85.60	5,089,659,000	1104	0	95.68
NT-PC-1	57,121,252	86.41	5,712,125,200	1522	0	95.08
NT-PC-2	49,432,496	87.13	4,943,249,600	1382	0	95.33
NT-PC-3	50,004,384	87.49	5,000,438,400	1488	0	95.38
Eth-24 h	57,160,978	86.81	5,716,097,800	1620	0	95.22
Eth-PC	69,577,816	87.70	6,957,781,600	1910	0	95.34
Eth-NC	47,402,348	85.38	4,740,234,800	1294	0	95.16
Total	381,595,864		38,159,586,400

Approximately 17,039 unigenes could be classified into three GO categories, namely, cellular component, biological process, and molecular function (Additional file 2). In addition, 12,650 and 5780 unigenes could be annotated according to UniProt and COG, respectively (Additional file 3). Around 4932 unigenes in the atemoya pericarp were mapped to 203 KEGG pathways (Additional file 4). The maps with the highest unigenes representation included metabolic pathways (map01100) and ribosome pathway (ko03010), followed by microbial metabolism in diverse environments (map01120), ribosome (map03010), plant hormone signal transduction (map04075), plant-pathogen interaction (map04626), spliceosome (map03040), RNA transport (map03013), protein processing in endoplasmic reticulum (map04141), ubiquitin-mediated proteolysis (map04120), and starch and sucrose metabolism (map00500) (Additional file 4).

### Screening and KEGG pathway enrichment analysis of differentially expressed genes (DEGs)

**Fig. 3:**
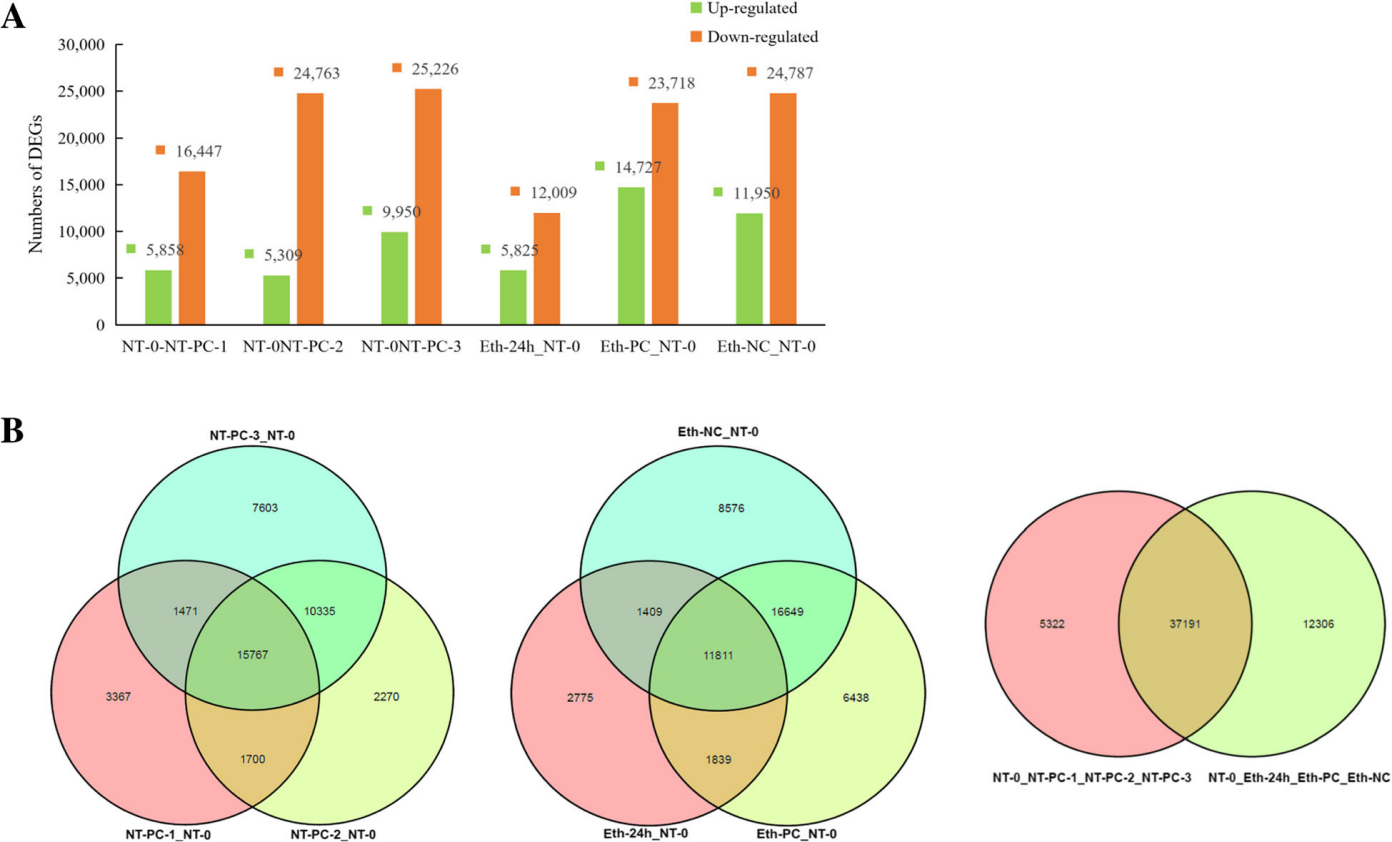
Expression profiling of cracking-related DEGs during atemoya fruit ripening. **a** The numbers of upregulated and downregulated DEGs revealed by paired comparison of the seven libraries. **b** a Venn diagrams representing the numbers of DEGs and the overlaps of sets obtained across three comparisons from two groups. Library form NT-0 pooled samples was used as calibrators to normalize the DEGs in other six libraries

We measured the unigene expression levels based on the obtained unique reads from seven libraries using RPKM values (Additional file 5), and then screened out DEGs using the combined criteria of FDR ≤ 0.05 and the |log2ratio| ≥ 1. The library generated from the pooled NT-0 samples was used to normalize the DEGs in the other six libraries, which was then followed by pairwise comparisons. Changes in gene expression were determined by comparing NT-PC-1/NT-0, NT-PC-2/NT-0, NT-PC-3/NT-0, Eth-24 h/NT-0, Eth-PC/NT-0, and Eth-NC/NT-0, respectively. NT-PC-1/NT-0, NT-PC-2/NT-0, and NT-PC-3/NT-0 were defined as the first group, and Eth-24 h/NT-0, Eth-PC/NT-0, and Eth-NC/NT-0 were designated as the second group. In these pairwise comparisons, we detected both unique and overlapping sets of DEGs. Figure 3a shows an enrichment of downregulated genes compared to upregulated genes during fruit softening and cracking. A total of 22,305, 30,072, and 35,176 DEGs were detected by analyzing NT-PC-1/NT-0, NT-PC-2/NT-0, and NT-PC-3/NT-0, respectively. A total of 42,513 DEGs, including 15,767 overlapping genes, were enriched during normal postharvest storage. For ethylene treatment, Eth-24 h/NT-0, Eth-PC/NT-0, and Eth-NC/NT-0, 49,497 DEGs showed enrichment, which included 11,811 overlapping genes. By comparing the first group and the second group, a total of 54,819 DEGs, including 5322 unique genes in the first group, 12,306 unique genes in the second group, and 37,191 overlapping genes were obtained (Fig. 3b).

**Fig. 4:**
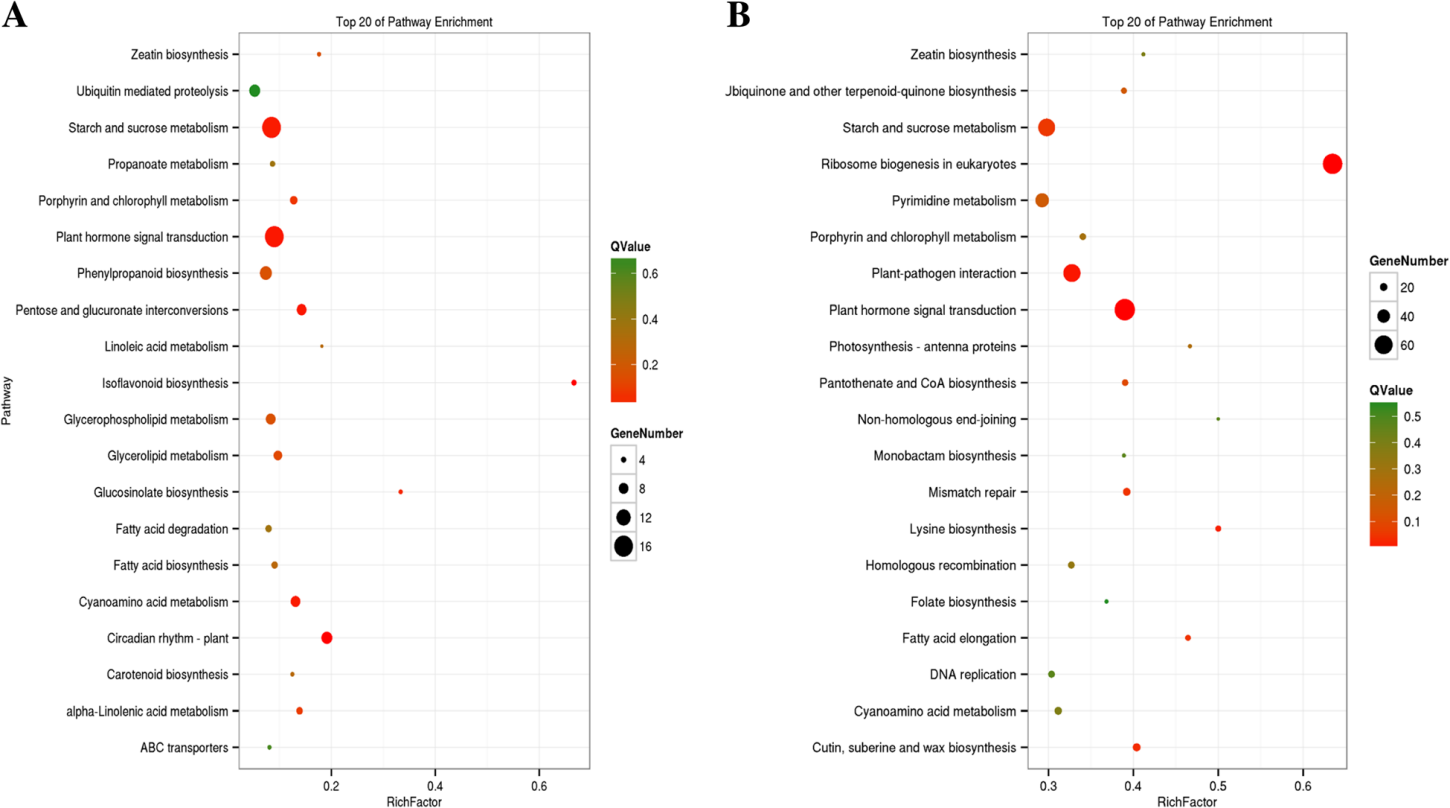
The top 20 KEGG pathways that are enriched with DEGs. **a** The KEGG pathways of up-regulated DEGs. **b** The KEGG pathways of downregulated DEGs. The greater dot indicates the more number of genes, and the deeper red color indicates the smaller Q-value, on the contrary the deeper green color indicates the bigger Q-value

Four libraries, including NT-0, NT-PC-1, NT-PC-2, and NT-PC-3, were selected and used in DEG profiling, and these libraries did not undergo treatment and exhibited cracking during fruit ripening. The number of up-and downregulated DEGs was determined by paired comparison of the four libraries, which was then followed by KEGG pathway enrichment analysis. The top 20 KEGG pathways with the highest number of up- and downregulated DEGs are shown in Fig. 4. Starch and sucrose metabolism (ko00500), plant hormone signal transduction (ko04075), pentose and glucuronate interconversions (ko00040), and cyanoamino acid metabolism (ko00460) pathways were significantly enriched with upregulated DEGs and showed lower Q values. The KEGG pathways of plant hormone signal transduction (ko04075), starch and sucrose metabolism (ko00500), plant-pathogen interactions (ko04626), and ribosome biogenesis in eukaryotes (ko03008) were enriched with downregulated DEGs. Among the top 20 KEGG pathways that were enriched with up- and downregulated DEGs, starch and sucrose metabolism (ko00500) and plant hormone signal transduction (ko04075) showed the highest number of DEGs and lowest Q-value, thereby suggesting that these two pathways play important roles during atemoya softening and cracking.

**Fig. 5:**
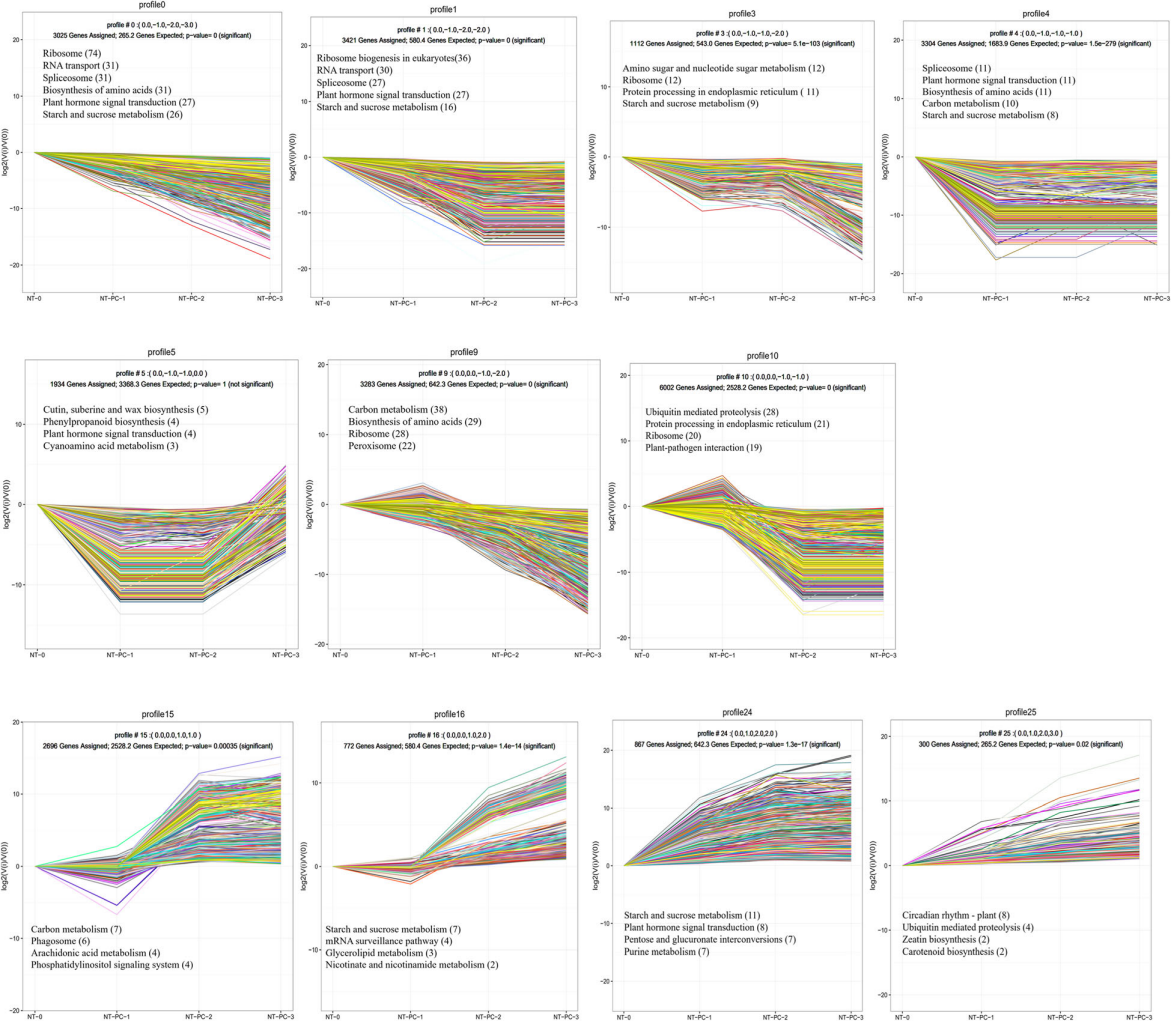
Cluster analysis of differentially expressed transcripts showing significant changes in expression profiles and KEGG pathway enrichment analysis. Enriched KEGG pathways are listed in each cluster. Numbers in brackets indicates the quantity of DEGs enriched

A total of 44,697 DEGs were obtained by pairwise comparison of NT-PC-1/NT-0, NT-PC-2/NT-0, NT-PC-3/NT-0, NT-PC-1/NT-PC-2, and NT-PC-2/NT-PC-3, and DEG expression profiles were analyzed by STEM. To gain further insights into the biological processes involved in fruit cracking, the DEGs were divided into 25 clusters, of which 11 represented distinct expression patterns. Figure 5 illustrates 11 clusters comprising 26,177 DEGs with significant differential expression (*P*-value < 0.05), including seven downregulated patterns (Profiles 0, 1, 2, 3, 4, 9, and 10) and four upregulated patterns (Profiles 15, 16, 24, and 25). The number of DEGs in each profile is shown in Additional file 6. A global description of the biological pathway enriched in each cluster of similarly regulated transcripts using KEGG pathway enrichment is presented in Fig. 5. The genes in clusters 0 and 1, including a broad range of genes responsible for “Ribosome”, “RNA transport”, “Spliceosome”, “Biosynthesis of amino acids”, and “Plant hormone signal transduction” were significantly downregulated from NT-0 to NT-PC-3. The genes from these two profiles mainly involved the degradation of materials in the basal metabolism pathway, which reflected the state of fruit maturation. Clusters 3, 4, and 5 contained genes that were initially downregulated and then subsequently upregulated. These findings suggest that these genes play roles in the later stages of storage (NT-PC-2 and NT-PC-3). These genes mostly belonged to the categories of Amino sugar and nucleotide sugar “metabolism”, “Ribosome”, “Protein processing in endoplasmic reticulum”, “Starch and sucrose metabolism”, “Spliceosom”, and “Plant hormone signal transduction”. The genes induced during the early stages of storage (NT-0 and NT-PC-1) and then subsequently downregulated were grouped into clusters 9 and 10. These genes were mainly involved in the “Carbon metabolism”, “Ubiquitin-mediated proteolysis”, “Ribosome” and “Peroxisome” pathways and might be responsible for the degradation of materials during earlier stages. Clusters 15, 16, 24, and 25 contained genes that were upregulated during the entire storage stage. These genes are involved in “Starch and sucrose metabolism”, “Carbon metabolism”, “Plant hormone signal transduction”, “Pentose and glucuronate interconversions”, and “Ubiquitin-mediated proteolysis”. The genes involved in “Starch and sucrose metabolism”, “Carbon metabolism”, and “Pentose and glucuronate interconversions” are all involved in sugar transformation, thereby confirming that sugar transformation plays an important role in atemoya fruit ripening and cracking, including the conversion of starch into soluble sugars and the degradation of soluble sugars into other forms to generate energy.

### Quantitative real-time PCR validation of DEGs

To further validate our RNA-seq results, we selected 16 unigenes for quantitative real-time PCR (RT-qPCR) analysis. In the KEGG enrichment pathway, the starch and sucrose metabolism pathway and plant hormone signal transduction pathway both ranked higher. Atemoya is the fruit of the starch storage type [25], and the degradation of starch and other sugars is an important metabolic process during ripening, which is closely related to fruit turgor pressure change [8]. In the present research, some cracking-related genes belong to cell wall polysaccharides, which are included in the starch and sucrose metabolism pathway. In addition, plant hormone signal transduction also has been associated with fruit cracking in some fruits, for example Litchi [26]. Thus, we selected 16 genes from these two metabolism pathways.

**Fig. 6:**
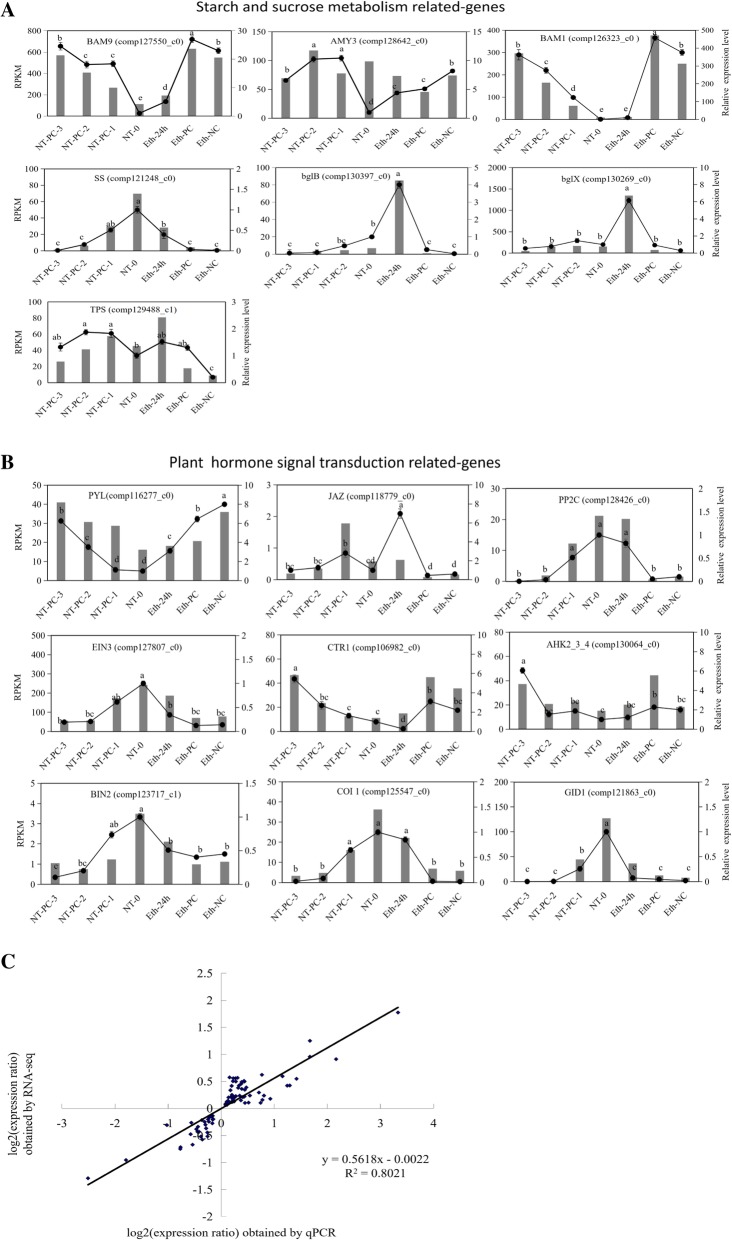
Candidate unigene expression levels verification and coefficient analysis of fold-change data between RT-qPCR and RNA-seq. **a** Transcript levels and RT-qPCR results of 7 selected genes from RNA-sequencing, which belonged to the starch and sucrose metabolism pathway. **b** Transcript levels and RT-qPCR results of nine selected genes from RNA-sequencing, which belonged to plant hormone metabolism pathway. The 16 unigenes were *BAM9*, *AMY3*, *BAM1*, *SS*, *blgB*, *blgX*, *COI1*, *TPS*, *GID1*, *PYL*, *JAZ*, *PP2C*, *EIN3*, *CTR1*, *AHK2–3-4*, and *BIN2*, and their full names are shown in the “Abbreviations” sections. The left y-axis shows the relative gene expression levels analyzed by qPCR (black lines). The right y-axis indicates the corresponding expression data of RNA-seq (gray histogram). The x-axis represents sampling time. The CT value of each gene was the average of three technical replicates with the standard error indicated. Significant difference of relative expression value in the different sampling times estimated by Duncan’s test was reported on the graphics (*p*-value < 0.05). Means labeled by the same letter are not significantly different. **c** Scatterplots were generated by the log2 expression ratios from RNA-seq (y-axis) and RT-qPCR (x-axis)

RNA samples from atemoya pericarp after ethylene treatment and the controls in normal temperature were used as templates. These unigenes are involved in starch and sucrose metabolism, including starch, cellulose, sucrose, and trehalose and plant hormone signal transduction pathways, including ethylene, cytokinin, gibberellin, brassiosteroids, abscisic acid, and jasmonic acid. In the seven sampling stages, the expression profiles of the candidate unigenes coincided with the results of sequencing (Fig. 6a and b) and RNA-seq analysis (Pearson correlation coefficients R2 = 0.8021; Fig. 6c), thereby validating our transcriptome analysis.

### Starch and sucrose metabolism pathway-related DEGs are involved in fruit ripening and cracking

**Fig. 7:**
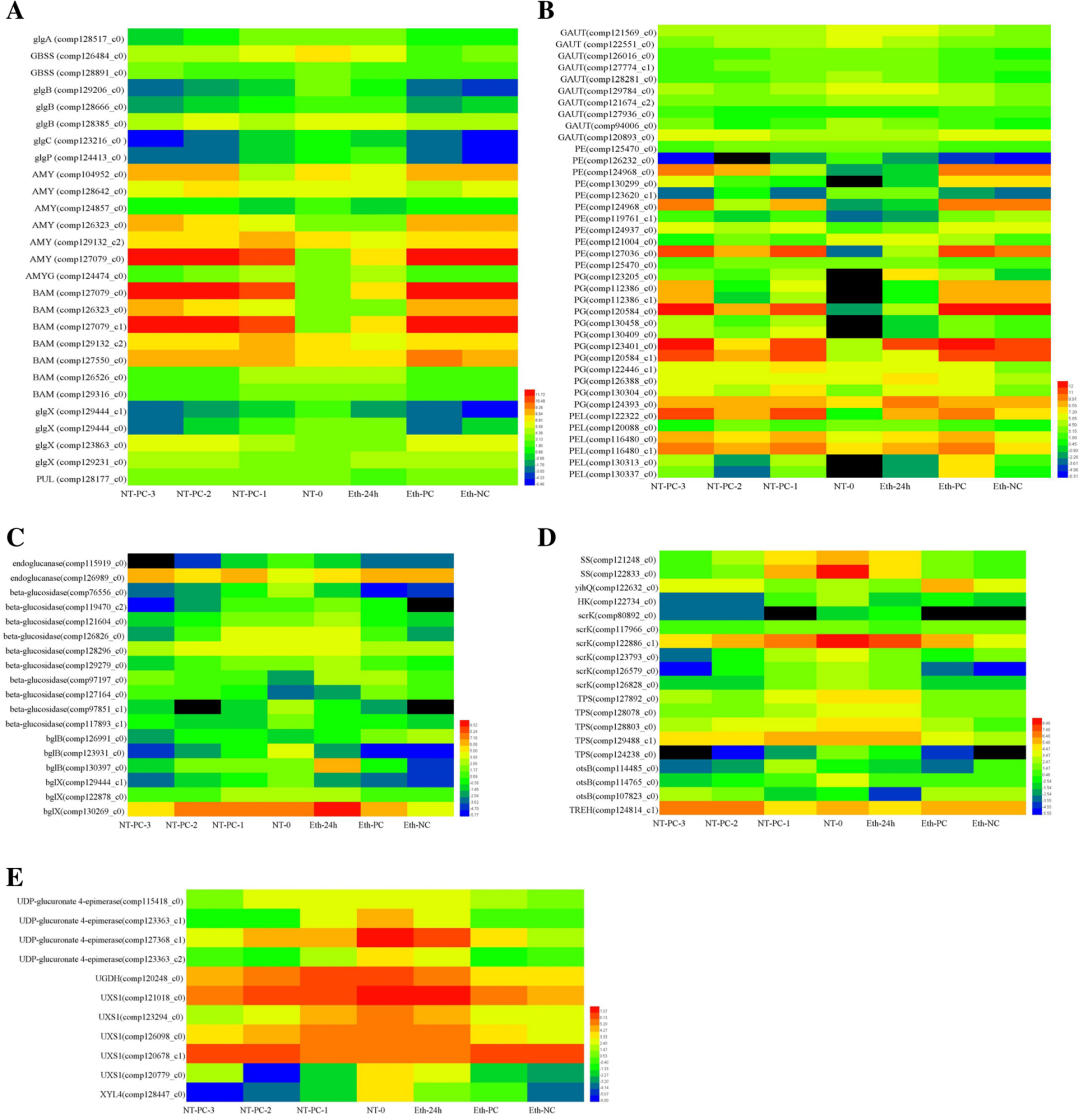
Heat map diagram of the expression patterns for DEGs annotated in the starch and sucrose metabolism pathway analyzed by KEGG. **a** Starch metabolism-related genes. **b** Pectin metabolism-related genes. **c** Cellulose metabolism-related genes. **d** Trehalose, sucrose, fructose, and glucose metabolism-related genes. **e** Other metabolism-related genes in the starch and sucrose metabolism pathway. Abbreviated names and full names of all of the genes in the heat map are listed in Table 2. The data on gene expression levels were normalized. Each column represents an experimental sample (e.g., NT-0, NT-PC-1, NT-PC-2, NT-PC-3, Eth-24 h, Eth-PC, and Eth-NC), and each row represents a gene. The color scale at the right represents the log-transformed RPKM value, and expression differences are shown in different colors. Red indicates high expression, and green represents low expression

KEGG pathway enrichment analysis of DEGs of each expression profile determined that the starch and sucrose metabolism pathway was significantly enriched in most clusters (Fig. 5). Table 2 shows the number of the DEGs from 11 differential expression clusters that were involved in the starch and sucrose metabolism pathway from NT-0 to NT-PC-3. A total of 39 DEGs were annotated in the starch and sucrose metabolism pathways, including starch, cellulose, pectin, sucrose, trehalose, maltose, glucose, fructose, and others. Approximately 114 unigenes were selected and used in heat map analysis (Fig. 7). The starch and sucrose metabolism pathway components are shown in Additional file 8.

**Table 2:** Numbers of DEGs involved in starch and sugar metabolism pathways during cracking of atemoya pericarp

	NT-0_NT-PC-1_NT-PC-2_NT-PC-3	
	Down regulation	Up regulation
	Profile 0, 1, 2, 3, 4, 9, 10	Profile 15, 16, 24, 25
Starch		
glgA; starch synthase [EC:2.4.1.21]	1	0
GBSS; granule-bound starch synthase [EC:2.4.1.242]	2	0
glgB; 1,4-alpha-glucan branching enzyme [EC:2.4.1.18]	3	0
glgC; glucose-1-phosphate adenylyltransferase [EC:2.7.7.27]	1	0
glgP; starch phosphorylase [EC:2.4.1.1]	1	0
AMYG; glucoamylase [EC:3.2.1.3]	1	0
AMY; alpha-amylase[EC 3.2.1.1]	2	4
BAM; beta-amylase [EC:3.2.1.2]	2	5
glgX; glycogen debranching enzyme [EC:3.2.1.196]	2	2
PUL; pullulanase [EC:3.2.1.41]	0	1
Cellulose		
endoglucanase [EC:3.2.1.4]	2	0
beta-glucosidase [EC:3.2.1.21]	8	2
bglB; beta-glucosidase [EC:3.2.1.21]	3	0
bglX; beta-glucosidase [EC:3.2.1.21]	2	1
Pectin		
GAUT; alpha-1,4-galacturonosyltransferase [EC:2.4.1.43]	7	3
PE; pectinesterase [EC:3.1.1.11]	3	8
PG; polygalacturonase [EC:3.2.1.15]	0	12
PEL; pectate lyase [EC:4.2.2.2]	1	5
Sucrose		
sucrose synthase [EC:2.4.1.13]	2	0
yihQ; alpha-glucosidase [EC:3.2.1.20]	0	1
Trehalose		
TPS; trehalose 6-phosphate synthase/phosphatase [EC:2.4.1.15 3.1.3.12]	8	0
otsA; trehalose 6-phosphate synthase [EC:2.4.1.15]	0	0
otsB; trehalose 6-phosphate phosphatase [EC:3.1.3.12]	1	2
TREH, treA, treF; alpha,alpha-trehalase [EC:3.2.1.28]	0	1
Maltose		
malQ; 4-alpha-glucanotransferase [EC:2.4.1.25]	0	3
Fructose		
scrK; fructokinase [EC:2.7.1.4]	9	0
HK; hexokinase [EC:2.7.1.1]	1	0
Glucose		
1,3-beta-glucan synthase [EC:2.4.1.34]	0	1
glucan 1,3-beta-glucosidase [EC:3.2.1.58]	0	1
Others		
UXS1, uxs; UDP-glucuronate decarboxylase [EC:4.1.1.35]	5	1
XYL4; beta-D-xylosidase 4 [EC:3.2.1.37]	2	0
UDP-glucuronate 4-epimerase [EC:5.1.3.6]	5	0
UGDH, ugd; UDPglucose 6-dehydrogenase [EC:1.1.1.22]	1	0
ENPP1_3; ectonucleotide pyrophosphatase/phosphodiesterase family member 1/3 [EC:3.1.4.1 3.6.1.9]	1	0
pmm-pgm; phosphomannomutase / phosphoglucomutase [EC:5.4.2.8 5.4.2.2]	0	2
pgmB; beta-phosphoglucomutase [EC:5.4.2.6]	2	1

In the starch metabolism pathway, there were four types of starch synthesis-related DEGs respectively encoding *glgA* (starch synthase), *glgB* (1,4-alpha-glucan branching enzyme), *glgC* (glucose-1-phosphate adenylyltransferase), and *GBSS* (granule-bound starch synthase). One *glgA*, 3 *glgB*, 1 *glgC*, and 2 *GBSS* were all downregulated from NT-0 to NT-PC-3 and after ethylene treatment during the entire storage period, whereas *GBSS* (comp126484_c0) was inhibited by ethylene compared to the control (Fig. 7a). Among the starch degradation-related unigenes, three types of unigenes respectively encoding *AMYG* (glucoamylase), *PUL* (pullulanase), and *glgP* (PYG starch phosphorylase) were enriched with a single gene (Table 2). Expression levels of *AMYG* and *glgP* slightly increased in NT-PC-1 and Eth-24 h, and then downregulated during the entire storage period, whereas *PUL* was upregulated both in normal storage conditions and after ethylene treatment (Fig. 7a). Two main types of starch-degrading enzymes encoding alpha-amylase (AMY) and beta-amylase (BAM) were enriched with six and seven unigenes, respectively (Fig. 7a and Table 2). There were four upregulated and two downregulated *AMY* unigenes, which exhibited similar expression patterns with normal storage conditions and after ethylene treatment. *AMY* (comp127079_c0) showed the highest expression levels, and *AMY* (comp104952_c0) was the second of six *AMY* unigenes that remains upregulated during the entire storage period, suggesting that these might play important roles in starch degradation. Seven *BAM* unigenes were enriched, of which five were upregulated, two were downregulated both with normal storage conditions and after ethylene treatment (Fig. 7a and Table 2). *BAM* (comp127079_c0) and *BAM* (comp127079_c1) had very high expression levels, higher than *AMY* (comp127079_c0) (Fig. 7a), whereas the two unigenes belonged to different fragments of the same gene, as indicated by BLAST. Compared to *AMY*, *BAM* might be the main enzyme of starch degradation. Four DEGs encoded *glgX* (glycogen-debranching enzyme), which included two downregulated and two upregulated genes both in normal storage conditions and after ethylene treatment. *GlgX* (comp129231_c0) induced higher expression with ethylene compared to the control. In the starch metabolism pathway, all of the synthetase genes were downregulated during fruit cracking, whereas most of the degradation-related genes, including *AMY*, *BAM*, *PUL*, and *glgX*, were upregulated. Together, these findings suggest that starch is extensively degraded during fruit ripening and cracking.

The pericarp is directly involved in fruit cracking, and the components of the pericarp are closely related to the development of cracking resistance. Most of the cell wall components are polysaccharides, such as cellulose and pectin. Enriched cellulose-related genes mainly encode cellulose-degrading enzymes, including two kinds of genes, namely, endoglucanase and beta-glucosidase. Two endoglucanase genes and 16 beta-glucosidase genes were enriched, of which 1 endoglucanase and 2 beta-glucosidase genes were upregulated and 1 endoglucanase and 14 beta-glucosidase were downregulated both with normal storage conditions and after ethylene treatment (Fig. 7c and Table 2). In 14 downregulated unigenes, the expression of 5 beta-glucosidase genes, including comp119470_c2, comp121604_c0,comp129279_c0,comp130397_c0, and comp130269_c0, were induced to different degrees using Eth-24 h, whereas these were continuously downregulated from NT-0 to NT-PC-3 with normal storage conditions (Fig. 7c).

An enrichment of pectin metabolism-related unigenes was also observed, except for *GAUT* (alpha-1,4- galacturonosyltransferase) that participates in pectin synthesis, as well as *PE* (pectinesterase), *PG* (polygalacturonase), and *PEL* (pectate lyase), which are involved in pectin degradation. Ten unigenes encoding *GAUT* were differentially expressed, of which three presented upregulated profiles, and the rest were downregulated both under normal storage conditions and after ethylene treatment. There were 11 *PE*, 12 *PG*, and 6 *PEL* unigenes that were respectively enriched, of which 8, 12, and 5 were upregulated both in normal storage conditions and after ethylene treatment (Fig. 7b and Table 2). Two *PE* unigenes (comp124968_c0 and comp127036_c0), three *PG* unigenes (comp120584_c0,comp123401_c0, and comp120584_c1), and two *PEL* unigenes (comp122322_c0 and comp116480_c1) presented higher expression than other pectin-degrading enzymes genes both under normal storage conditions and after ethylene treatment, but there were no obvious differences between the controls and ethylene treatment (Fig. 7b). An enrichment of pectin-degrading enzymes was also observed, and most of these were upregulated, which suggested that pectin degradation was intimately linked to fruit ripening and cracking. Several unigenes encoding *TPS* (trehalose 6-phosphate synthase/phosphatase), *malQ* (4-alpha-glucanotransferase), *scrK* (fructokinase), *SS* (sucrose synthase), 1,3-beta-glucan synthase, *UXS1* (UDP-glucuronate decarboxylase), and other sugar metabolism-related genes are involved in trehalose, maltose, sucrose, fructose, and glucose metabolism pathways, which ware responsible for converting all kinds of polysaccharides into monosaccharides. The contribution of different kinds of sugars to osmotic pressure varies, with monosaccharides providing the largest contribution, and the rate of conversion of polysaccharides to monosaccharides can influence osmotic pressure. Therefore, enrichment analysis of sugar-related genes may identify the major sugars that are metabolized in atemoya fruits and their possible effects on changes in osmotic pressure during fruit cracking. According to enriched sugar related-genes, trehalose, sucrose, fructose, glucose, and maltose are the major soluble sugars in sugar metabolism during fruit ripening. Most of these were downregulated during the entire storage period; nevertheless, some of these had high expression levels at NT-0, NT-PC-1 and Eth-24 h, e.g., *SS* (comp122833_c0), *scrK* (comp122886_c1), *TREH* (alpha,alpha-trehalase)(comp124814_c1), UDP-glucuronate 4-epimerase (comp127368_c1), and *UXS1* (comp121018_c0) (Fig. 7d and e). The above results only preliminarily identified the major types of sugar, and the next step is to quantify the contents of the major sugars at each stage to determine the conversion rate of sugars and their effect on osmotic pressure.

### Changes in starch and pectin contents and their metabolism-related genes expression profiles

**Fig. 8:**
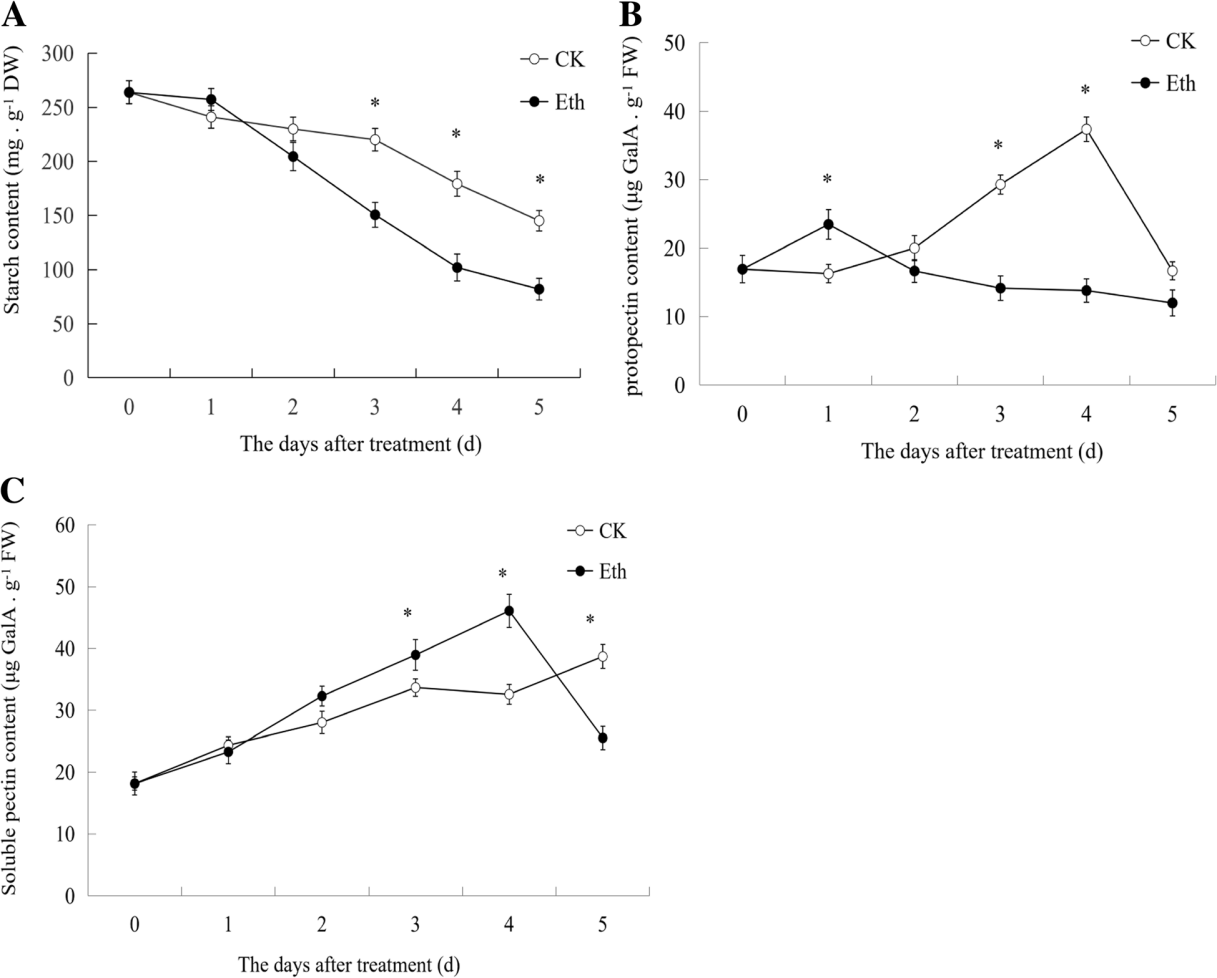
Analysis of starch, protopectin, and soluble pectin contents with or without ethylene treatment during atemoya storage. **a** Starch (**b**) Protopectin (**c**) Soluble pectin. Vertical bars represent the standard error of three biological replicates. Significant differences in starch, protopectin, and soluble pectin content for each day between no treatment (CK) and ethylene treatment (Eth) estimated using the t-test is shown in the graph (p-value < 0.05). Asterisks (*) indicate significant differences in starch, protopectin, and soluble pectin content between CK and Eth at the same day

Figure 8a shows that starch content decreased during fruit storage, and ethylene treatment accelerated starch degradation. Protopectin content in the pericarp without treatment increased until day 4, whereas it decreased after day 1 with ethylene treatment (Fig. 8b), which indicated that ethylene inhibited the synthesis of the protopectin. Figure 8c shows that soluble pectin content increased during fruit storage, and ethylene treatment enhanced soluble pectin content compared to no treatment, which suggests that ethylene treatment accelerates pectin degradation.

**Fig. 9:**
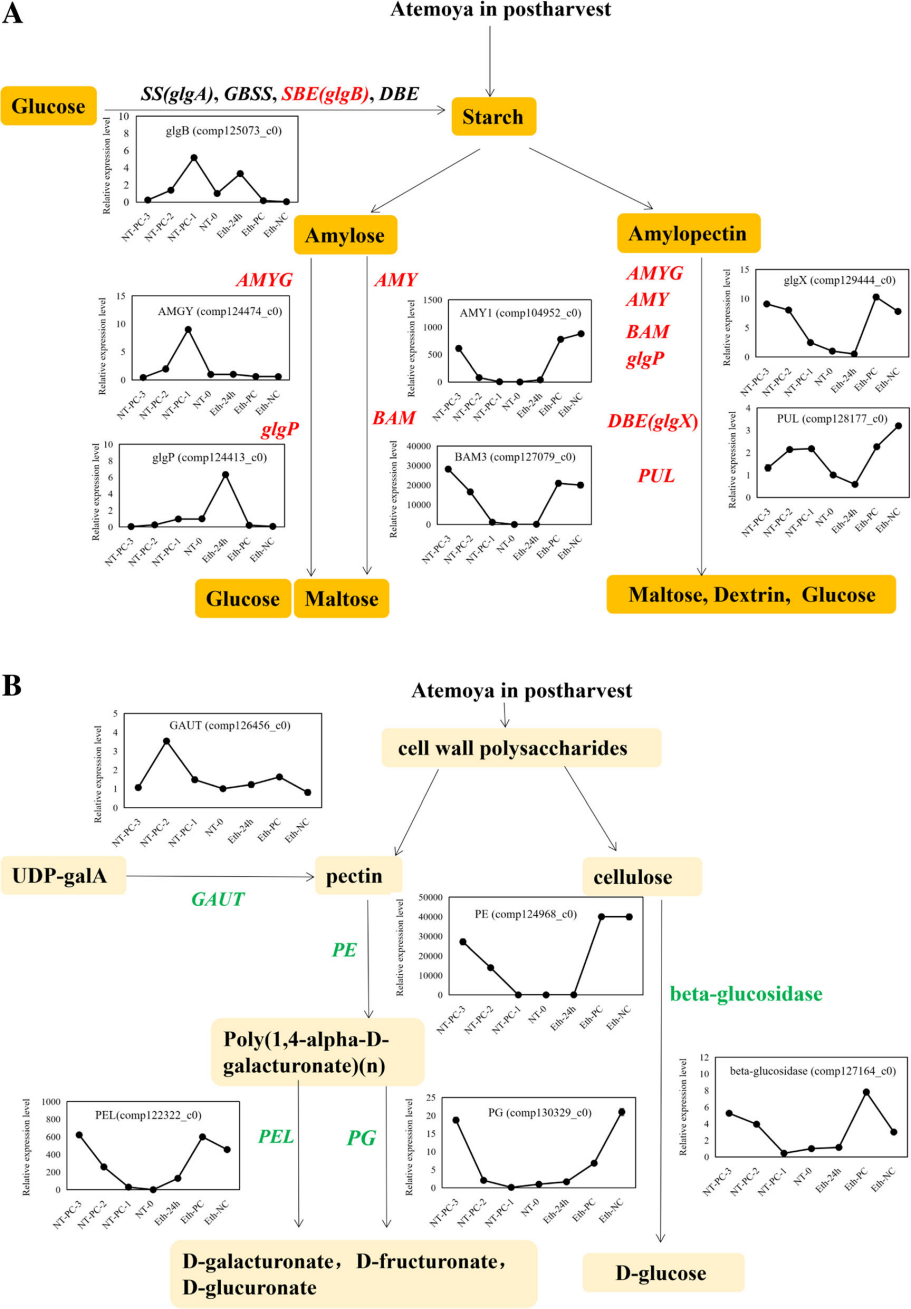
Simplified scheme of the polysaccharide metabolism pathway. (A) Starch metabolism pathway; genes examined are in red italic bold letters, which include *glgB*, *AMY1*, *BAM3*, *AMYG*, *glgP*, *glgX*, and *PUL*.(B) Pectin and cellulose metabolism pathway. Genes examined are in green italic bold letters, which include *GATU*, *PE*, *PG*, *PEL*, and *beta-glucosidase*. The full names of the genes are shown in Table 2 and the part of the “Abbreviations” section

To shed light on which genes play important roles in starch and cell wall polysaccharide metabolism pathways during fruit cracking, the expression profiles of 12 unigenes were analyzed. Among these, *AMY3* (comp128642_c0), *BAM1* (comp126323_c0), and *BAM9* (comp127550_c0) that were validated by RT-qPCR (Fig. 6a). Nine starch-degrading enzymes, namely, *AMY1* (comp104952_c0), *AMYG* (comp124474_c0), *BAM3* (comp127079_c0), *PUL* (comp128177_c0), *glgX* (comp129444_c0), *glgP* (comp124413_c0), *AMY3*, *BAM1*, *BAM9*, and one starch synthesis gene *glgB* (comp125073_c0), were selected (Fig. 9a). Among these, except for *glgP* and *AMYG*, the expression of the other degrading enzyme genes were significantly upregulated, whereas that of the synthesis genes decreased during fruit cracking after ethylene treatment and in the controls. *AMY1*, *BAM3*, *BAM9*, *PUL*, and *glgX* were all upregulated and exhibited higher expression levels with ethylene treatment compared to without treatment, which could be responsible for accelerating starch degradation after ethylene treatment. With ethylene treatment and in the controls, *AMY1* and *BAM3* were significantly expressed, which indicated these two genes are the key genes of starch degradation.

*GAUT* is the key pectin synthesis enzyme [27]. High amounts of *GAUT* (comp126456_c0) accumulated during the first three stages, with a reduction at the fourth stage with controls and ethylene treatments, and higher expression levels under controls compared to ethylene treatment, whereas its expression profile coincided with changes in protopectin content, which could account for protopectin synthesis during fruit cracking and softening (Fig. 9b). *PE* (comp124968_c0), *PG* (comp130329_c0), and *PEL* (comp122322_c0) were upregulated with or without ethylene treatment, which indicated that these three genes play important roles in pectin degradation. Among these, *PE* was significantly upregulated by ethylene, and thus might be the key gene involved in pectin degradation (Fig. 9b). *Beta-glucosidase* (comp127164_c0) was upregulated by ethylene, indicating that it could be a major cellulose degradation enzyme (Fig. 9b).

## Discussion

Several factors influence fruit cracking, including genetics, morphology, physiology, environmental conditions (e.g., temperature, wind and light), and orchard management (e.g., irrigation and nutrition). Furthermore, fruit cracking is a quantitative trait that is controlled by several genes [8]. To generate molecular evidence and elucidate the mechanism underlying fruit cracking, the present study attempted to identify fruit cracking genes in atemoya during postharvest storage. To circumvent the unavailability of an atemoya reference genome, we utilized the pericarp of cracking atemoya fruits for RNA-seq in the de novo assembly of a reference transcriptome. Our assembly contains a total of 64,069 unigenes with a mean size of 670 bp. Approximately 25,074 unigenes were annotated to public protein databases. We performed a pairwise comparison and identified 42,513 DEGs between NT-0 and NT-PC-1, NT-PC-2, and NT-PC-3 and 49,497 DEGs between NT-0 and Eth-24 h, Eth-PC, and Eth-NC. KEGG pathway enrichment analysis of all DEGs and DEGs of each distinct expression patterns indicated that the starch and sucrose metabolism pathway was significantly enriched.

The present study determined that in cherimoya, carbohydrates are mainly stored in the form of starch [28]. Scanning electron microscope (SEM) analysis of AP atemoya indicated numerous starch granules in the fruit tissues [29]. Thus, the cells of the atemoya fruit not only store starch but also use this to maintain cell turgor [30]. Mo et al. [11] suggested that the firmness of Annona fruits dramatically decreases with ripening, possibly due to transformation of starch into soluble sugars. Wills et al. [31] showed that the initiation of cracking coincides with the decline in about one-fifth of the starch level in fruits. Paull [14] suggested that atemoya cracking commonly occurs during postharvest because of changes in osmotic and subsequent turgor, which are related to the production of neutral sugars during ripening, which leads to the movement of water from the skin and receptacle to the flesh. Previous studies have revealed that starch degradation induces changes in fruit turgor, and fruit softening and cracking are closely related; however, these were mainly observed at the physiological level. In the present study, transcriptome sequencing revealed that fruit cracking is strongly associated with a significant enrichment of several starch metabolism genes, including 7 starch synthase genes and 17 starch degrading enzymes. At the transcriptional level, the transformation of starch to soluble sugars may be one of the most important processes in fruit softening and cracking. Unigenes encoding starch synthase were all downregulated, whereas starch degradation genes were mostly upregulated. For example, *AMY1* (comp104952_c0) and *BAM3* (comp127079_c0) were significantly induced and highly expressed during fruit cracking, which suggests that these are key to starch degradation, and simultaneously showed that starch is rapidly decomposed by amylase during postharvest. We also showed that starch degradation is related to fruit cracking, which coincides with the results of previous studies on physiological metabolism.

Soluble sugar content affects fruit turgor, thereby leading to fruit cracking. Previous studies on sweet cherry, wax apple, litchi, grapes, and other fruits have confirmed that the soluble sugar content is higher and osmotic potential is lower in cracking fruits than those without cracking [20, 32–34], which indicates that water, soluble sugar, and osmotic potential are closely related to fruit cracking. Lu and Lin [20] reported that the TSS content and total titratable acid levels were both 20% higher in cracked fruits than in non-cracked fruits of wax apple. Furthermore, when its turgor pressure was 60% higher, an increase in TSS content and total titratable acid levels during fruit maturation was observed, which in turn leads to a decrease in tissue osmotic potential. Considine and Kriedemann [32] provided direct evidence for the importance of fruit soluble solids, wherein cracking in grapes occurs when these are submerged in solutions of low osmotic potential. Atemoya fruits are of the starch storage type [25], and the rate and ability to undergo starch degradation during postharvest is directly related to soluble sugar content, which in turn directly affects fruit turgor [8]. Therefore, factors influencing starch degradation rate, such as temperature and ethylene, could also influence the degree of fruit cracking. Under low temperature, TSS content of cherimoya fruits slowly increased, whereas no detectable change in pH value was observed [35, 36], which suggests that low temperature treatment of cherimoya results in a decrease in the rate of starch hydrolysis and monosaccharide concentration, and these findings agree with the results of our investigation in atemoya [37]. Gutiérrez et al. [28] determined using ultrastructural analysis of fruits that starch grains do not break down, and the cell walls remain intact after 6 d of storage at 4 °C. Except for low temperature, our previous research suggests that ethylene could significantly accelerate cracking and ripening in atemoya, and the pericarp cracks first after 2 g/kg of ethephon treatment, which is different from the control treatment [22]. This research found that ethylene accelerated starch degradation and enhanced the content of soluble pectin, compared to the control, suggesting that ethylene accelerates fruit cracking and ripening. Transcriptome analysis suggests a strong association between the degradation of starch, pectin, and cellulose, and the upregulation of *AMY1* (comp104952_c0), *BAM3* (comp127079_c0), *BAM9* (comp127550_c0), *PUL* (comp128177_c0), *glgX* (comp129444_c0), *PE* (comp124968_c0) and *beta-glucosidase* (comp127164_c0). In addition, the ethylene treatment not only promoted the increase of soluble pectin, but also inhibited the synthesis of the propectin, which could explain why the pericarp cracks first after ethylene treatment. At the transcriptional level, *GAUT* (comp126456_c0) is mainly involved in the synthesis of the propectin. The above research showed that the external environment temperature and ethylene treatment could be directly related to the time and extent of fruit cracking by regulating the metabolism of starch and cell wall polysaccharides.

Previous studies have found that starch is transformed into various kinds of soluble sugars such as disaccharides (e.g., sucrose) and monosaccharides (e.g., fructose and glucose). Paull et al. [38] reported that in soursop, sucrose content increased by four-fold at maturity, and the highest concentration was observed at 3 days after harvest, which subsequently decreased to 40%. Fructose and glucose content then slowly increase until 5 days after harvest. The ratio of sucrose, glucose, and fructose at the edible ripe stage was 4.3:3.0:3.2 [39]. However, in cherimoya (*Annona cherimola* Mill. cv. ‘Fino de Jete’), starch is mainly converted into glucose and fructose in equimolar concentrations, with little and transient accumulation of sucrose [28, 40]. In our research, some genes that were involved with sucrose, fructose, and glucose were enriched, such as sucrose-metabolizing enzymes, *SS* and alpha-glucosidase (*YihQ*), and fructose metabolism-related enzymes, *scrK*, and hexokinase (*HK*), glucose metabolism genes, and glucan 1,3-beta-glucosidase. Furthermore, trehalose metabolism-related genes, including eight trehalose 6-phosphate synthase (*TPS*) genes, three trehalose 6-phosphate phosphatase (*otsB*) genes, one *TREH* gene, and three maltose metabolism-related genes *malQ*, were enriched. The degradation of starch produces maltose, whereas previous studies have shown that trehalose is rarely involved in starch transformation of Annona fruits. Recent studies have observed that trehalose in plants is also involved in metabolism and gene expression regulation and affects plant growth, development, and responses to changes in the external environment [41, 42]. Trehalose is a typical stress metabolism and protective substance, and when the organism is under stress, it can quickly accumulate trehalose [43, 44]. The role of trehalose during atemoya postharvest requires further investigation.

Researchers have found that some cell wall-related genes, which include *EXP*, *XET*, *PG*, and *PE*, also play roles in fruit cracking [8, 21]. Shen et al. [19] suggested that during cracking and ripening of fruits in the field, the expression of *AsEXP1* and *AsEXP3* are relatively high, whereas that of *AsEXP2* is relatively weak. Wang et al. [17] reported that the differential expression of LcEXP1 and LcEXP2 in the litchi pericarp is related to fruit growth and resistance to crack formation. Our previous research [22] compared cracking-susceptible cultivars with cracking- resistant cultivars of atemoya during postharvest storage and we found that EXP1 is more relevant to fruit maturity and softening. *EXP2*, *EXP3*, and *PE* were more closely related to fruit cracking, whereas *XET1*, *XET2*, and *XET3* might be related to fruit softening and are unlikely key genes of fruit cracking [22]. Lu and Lin [20] reported a 131% increase in PG activity in cracked fruits compared to non-cracked wax apple fruits. In the present research, transcriptome sequencing analysis found that except for 12 *PG* and 11 *PE*, another pectin degradation-related gene, 6 *PEL*, was enriched, suggesting that pectin decomposition is also an important polysaccharide metabolism process in atemoya fruit cracking. At the physiological level, combining part of the gene expression evidences from previous researches with our RNA-seq results strengthens our theory that atemoya fruit cracking is caused by the transformation of starch into soluble sugars that in turn increases turgor pressure, thereby resulting in the rupture of cells and tissues, as well as the degradation of cell wall polysaccharides such as pectin and cellulose, ultimately decreasing cell wall toughness, which is exhibited as cracking of the pericarp and flesh.

RT-qPCR analysis indicated an enrichment of numerous starch, pectin, and cellulose metabolism-related genes, which were predominated by *AMY1* (comp104952_c0), *BAM3* (comp127079_c0), *PUL* (comp128177_c0), *glgX* (comp129444_c0), *PE* (comp124968_c0), *PG* (comp130329_c0), *PEL* (comp122322_c0), and *beta-glucosidase* (comp127164_c0). Since the present study was designed as a preliminary validation, additional studies looking into the kind of relationship between these genes and fruit cracking are warranted. In addition, fruit cracking is a complex physiological phenomenon in that no single gene directly controls the process. However, transcriptome sequencing has provided molecular evidence that starch and cell wall polysaccharides may both be involved in fruit cracking, as well as useful genetic resources for screening additional fruit cracking-related genes.

In addition to the important role of starch and sucrose metabolism pathway in atemoya fruit cracking, studies have shown that plant hormone signal transduction pathways are also involved in fruit cracking. Spraying different concentrations of gibberellin on cherries, pomegranates, and litchi can reduce fruit cracking rate [45–47]. In litchi and pomegranate, ABA content in pericarp is higher in cracking fruits than in the healthy fruits [48, 49], and spraying ABA increases the fruit cracking rate of litchi [11]. These researchers suggest that spraying GA can reduce fruit cracking, but spraying ABA can increase fruit cracking in some fruits. Li et al. [26] performed comparative transcriptomic analyses of non-cracking and cracking litchi fruits and found that plant hormone signal transduction pathway-related genes have been enriched, including GA and ABA. They believe that lower GA levels and higher ABA levels in pericarp lead to slower pressure expansion of the pericarp, so that aril cells produce more turgor against the skin, thereby leading to fruit cracking. In our results, the enriched number of DEGs of the plant hormone signal transduction pathway ranked fourth in all metabolism pathways, consisting of a total of 154 DEGs, including gibberellin, abscisic acid, and cytokinins (Additional 4), whereas we did not focus on plant hormone-related genes in this study, and thus their role in fruit cracking requires further investigation.

## Conclusions

In summary, seven sets of transcriptome data comprising 64,069 unigenes in the atemoya pericarp were generated by Illumina sequencing. Both KEGG pathway enrichment analysis of DEGs and material content analysis confirmed that starch decomposition into soluble sugars and cell wall polysaccharides metabolism are closely related to the ripening and cracking of the AP atemoya. RT-PCR analysis indicated that the differential expression of genes during different cracking stages of the atemoya fruit influences starch and pectin content. In addition, plant hormone signal transduction pathway-related genes were significantly enriched, suggesting their role in fruit ripening and cracking. The present study may serve as a valuable resource for future atemoya genomic studies and may also benefit investigations involving other closely related agricultural species. The DEG dataset may also provide useful candidate genes for the elucidation of the mechanism underlying fruit cracking in atemoya and other fruits.

## Methods

### Plant materials and experimental design

In the present study, AP atemoya fruits were collected from the Base of National Field Genebank for Tropical Fruit at South Subtropical Crops Research Institute, Chinese Academy of Tropical Agricultural Sciences, Zhanjiang, Guangdong, China. The orchard is located at 21°12′ N,110°4′E. Material collection was conducted in accordance with local legislation, and there was no need for permission from other organizations. We complied with the Convention on the Trade in Endangered Species of Wild Fauna and Flora. Fruits were harvested in October, when fruit skin color changed from darker to lighter green and can be greenish yellow, and those free of blemishes and mechanical damage were collected [14].

The first sublots of fruits were used for transcriptome sequencing. The atemoya fruits were picked at about 80% ripening. Twenty fruits were kept at room temperature with no treatment, and recently picked fruits were defined as day 0 or NT-0. Most of the fruits without any treatment started to crack at the pedicel on day 2 and were named NT-PC-1, whereas splits increased and deepened on day 3 (NT-PC-2) and day 4 (NT-PC-2) with prolonged storage time. The following four stages were selected for transcriptome sequencing: recently picked fruits (NT-0), cracking at the pedicel (NT-PC-1), moderate cracking at the pedicel (NT-PC-2), and severe cracking at the pedicel (NT-PC-3). Another 20 fruits were immersed in 2 g/kg ethephon for 2 min, transferred into a closed plastic box, and the lid was left open after 24 h. Fruits that did not exhibit cracking after 24 h of ethephon treatment were designated as Eth-24 h. Most fruits initially exhibited cracking in the pericarp on day 2 and were named Eth-PC. On day 3, the fruits showed more extensive and deeper cracking, including the pericarp and the peduncle. However, not all of the fruits after ethephon treatment appeared cracking, and no-cracking fruits were defined as Eth-NC on day 4 after ethephon treatment. Samples were collected from three stages for transcriptome sequencing, including fruits 24 h after ethephon treatment (Eth-24 h), fruits with pericarp cracking after ethephon treatment (Eth-PC), and no fruit cracking after ethephon treatment (Eth-NC).

Three pericarp samples that showed similar TSS contents, firmness values, and degree of cracking under the same treatment at each stage were collected, immediately frozen in liquid nitrogen, and then stored at − 80 °C until further processing. Three replicate samples were prepared for each biological sample. The pericarps of the candidate samples, including NT-0, NT-PC-1, NT-PC-2, NT-PC-3, Eth-24 h, Eth-PC, and Eth-NC, were subjected to transcriptome sequencing and expression analysis.

The second sublots of fruits were used for starch and pectin content determination. Fifteen fruits were stored at room temperature with no treatment, and another 15 fruits were treated with ethephon as earlier described. Samples were collected on days 0, 1, 3, and 5. Three pericarp samples at each stage were collected, immediately frozen in liquid nitrogen, and then stored at − 80 °C until further processing.

### Total soluble solids (TSS), firmness value, starch, and pectin determination

TSS content and firmness were measured as previously described [22]. A solution of 80% Ca (NO_3_)_2_ was used to extract starch from the ethanol-insoluble residue, and the absorption value of the reaction product at a wavelength of 620 nm was determined using the iodine-iodide kalium method according to the method of Cao et al. [50]. A solution of 95% ethanol was used to extract pectin in a boiling water bath. The supernatant was used to determine soluble pectin, and the ethanol-insoluble residue hydrolyzed by sulfuric acid was used to measure protopectin content according to the method Cao et al. [50]. Pectin was determined by carbazole, and the content of pectin was indicated by the quality of galacturonic acid.

### RNA extraction and illumina sequencing for transcriptome analysis

Seven libraries (NT-0, NT-PC-1, NT-PC-2, NT-PC-3, Eth-24 h, Eth-PC, and Eth-NC) were designed for RNA- Seq to obtain a general overview of the atemoya pericarp transcriptome of fruit ripening and cracking. Total RNA was extracted from pericarp tissues using a Quick RNA isolation kit following the manufacturer’s instructions (Huayueyang, China). The RNA samples were digested with DNase I to remove possible contaminating genomic DNA and purified with RNase-free columns (Huayueyang, China). RNA quality was verified using a 2100 Bioanalyzer RNA Nanochip (Agilent, Santa Clara, CA, USA), and RNA integrity was confirmed by electrophoresis on formaldehyde-containing 1.5% (*w*/*v*) agarose gels. Then, RNA was quantified using a NanoDrop ND-1000 spectrophotometer (Nano-Drop, Wilmington, DE, USA). Three high-quality RNAs were pooled by mixing equal quantities of RNA of the three biological replicates used in RNA-seq at each stage. Each library was sequenced in an Illumina HiSeq 2500 system using 100-bp paired-end protocols. All of the raw transcriptome data were deposited to the GenBank Short Read Archive (Accession number SRP114346).

### De novo assembly and functional annotation

The resulting RNA sequencing data were subjected to purification, which included removing adapters and low-quality reads. Clean reads were de novo assembled using the Trinity Program [51]. The resulting sequences obtained with Trinity were then called unigenes. To annotate the unigenes, we used the BLASTx program (http://www.ncbi.nlm.nih.gov/BLAST/) of NCBI with an E-value threshold of 1e− 5 to the NCBI non-redundant protein (Nr) database (http://www.ncbi.nlm.nih.gov), the Universal Protein Resource (UniProt) database (http://www.uniprot.org/), the Kyoto Encyclopedia of Genes and Genomes (KEGG) database (http://www.genome.jp/kegg), the Gene Ontology (GO) database (http://geneontology.org/), and the Cluster of Orthologous Groups of protein (COG) database (http://www.ncbi.nlm.nih.gov/COG). Unigene expression level was normalized by calculating reads per kb per million reads (RPKM) [52]. Comparison of unigene expression among treatments was according to DEGSeq as described by Anders and Huber [53] and Wang et al. [54]. DEGs between treated and control samples were identified when the false discovery rate (FDR) ≤ 0.05, |log2ratio| ≥ 1, and *P*-value < 0.05. The library prepared from the NT-0 pooled samples was used as calibrators to normalize the DEGs in the other six libraries (NT-PC-1, NT-PC-2, NT-PC-3, Eth-24 h, Eth-PC, and Eth-NC). DEGs were clustered by Short Time-series Expression Miner software (STEM) [55]. The clustered profiles with a P-value ≤0.05 were considered to be significantly expressed. Then the DEGs were subjected to KEGG pathway annotation using Blast all software against the KEGG database. KEGG pathways with a Q-value ≤0.05 are significantly enriched in DEGs. Senior bubble charts were constructed using the OmicShare tools, a free online platform for data analysis (www.omicshare.com/tools). To understand the gene expression patterns of DEGs during fruit cracking, we performed a hierarchical cluster analysis of expression patterns with cluster software [56] and Java TreeView [57].

### RT-qPCR validation

RT-qPCR was applied to investigate gene expression patterns. First-strand cDNA was generated from 1 μg total RNA isolated from the seven pericarp samples using the PrimeScript™ RT reagent kit with gDNA Eraser (TaKaRa, Japan). RT-qPCR primers for were designed using Primer Premier 5.0 software (Premier, Canada) and synthesized by Sangon Biotech (Shanghai, China) Co., Ltd. The atemoya homologue actin (GenBank Accession Number MF893339) was selected as reference. All of the primers of the candidate unigenes are shown in Additional file 7. Each reaction (final volume: 10 μL) contained 5 μL of 2× SYBR Green mastermix (Thermo Fisher), 1 μL of each of the forward and reverse primers (0.25 mM), 1 μL of the cDNA template, and 2 μL of RNase-free water. RT-qPCR was conducted in a LightCycler 480 system (Roche, USA) using the following conditions: 95 °C for 5 min, followed by 40 cycles of 95 °C for 30 s, 55 °C for 30 s, and 72 °C for 30 s in 96-well optical reaction plates. Each RT-qPCR analysis was performed in triplicate. Expression levels of the tested reference genes were determined by CT values and calculated using 2^-△△Ct^ [58].

### Statistical analysis

All of the measurements were repeated thrice. All of the data were subjected to ANOVA according to the model for a completely randomized design using SPSS software (SPSS, Inc., Chicago, IL, USA). Differences between two sample evaluated by *t* test at the 0.05 level in starch and pectin determination and differences among more samples were evaluated by Duncan’s test at the 0.05 level in RT-qPCR analysis.

## Additional files

Additional file 1: **Figure S1.** Size distributions of unigenes in the reference library. (DOC 21 kb)

Additional file 2: **Figure S2.** GO assignment of all of the unigenes in the reference Library. (DOC 195 kb)

Additional file 3: **Figure S3.** COG assignment of all of the unigenes in the reference library. (DOC 55 kb)

Additional file 4: **Table S1.** Pathway annotation of unigenes and the number of pathway from cracking atemoya pericarp. (XLSX 162 kb)

Additional file 5: **Table S2.** RPKM of the all of the unigenes in cracking pericarp of atemoya. (XLS 11116 kb)

Additional file 6: **Figure S4.** Profiles ordered according to *P*-value significance of the number assigned versus expected. Numbers between brackets indicate the number of the DEGs assigned. (DOC 2023 kb)

Additional file 7: **Table S3.** Primer sequences of the reference gene and candidate unigenes used in RT-qPCR analysis. (XLS 64 kb)

Additional file 8: **Figure S5.** Enriched starch and sucrose metabolism pathway. The components marked with red rectangles were considered differentially expressed. (DOC 32 kb)

## Abbreviations

1-MCP: 1-methylcyclopropene
AMY: Alpha-amylase
AMYG: Glucoamylase
BAM: Beta-amylase
bglB: Beta-glucosidase
bglX: Beta-glucosidase
COI1: Coronatine-insensitive protein 1
CTR1: Serine/threonine-protein kinase
DBE: Starch debranching enzyme
DEGs: Differentially expressed genes
EIN2: Ethylene-insensitive protein 2
EIN3: Ethylene-insensitive protein 3
EXP: Expansins
GBSS: Granule-bound starch synthase
GID1: Gibberellin receptor GID1
glgA: Starch synthase
glgC: Glucose-1-phosphate adenylyltransferase
glgP: Starch phosphorylase
glgX: Glycogen debranching enzyme
HK: Hexokinase
JAZ: Jasmonate ZIM domain-containing protein
malQ: 4-alpha-glucanotransferase genes
otsB: Trehalose 6-phosphate phosphatase
PE: Pectinesterase
PEL: Pectate lyase
PG: Polygalacturonase
PP2C: Protein phosphatase 2C
PUL: Pullulanase
PYL: Abscisic acid receptor PYR/PYL family
RT-qPCR: Reverse transcription-quantitative real time PCR
SBE: Starch branching enzyme
scrK: Fructokinase
SS: Starch synthase
TPS: Trehalose 6-phosphate synthase/phosphatase
TREH: Alpha, alpha-trehalase
XET: Xyloglucan endotransglycosylase
YihQ: Alpha-glucosidase
AHK2_3_4: Arabidopsis histidine kinase 2/3/4
GAUT: Alpha-1,4- galacturonosyltransferase
glgB: 1,4-alpha-glucan branching enzyme

## Authors’ information

All authors works/study in the South Subtropical Crops Research Institute, Chinese Academy of Tropical Agricultural Sciences, Zhanjiang, 524091, China.
