# Supplementary figures and images for: Transcriptome analysis of atemoya pericarp elucidates the role of polysaccharide metabolism in fruit ripening and cracking after harvest
Source: BMC Plant Biol. 2019 May 27;19:219. doi: 10.1186/s12870-019-1756-4 (PMC6537181; doi:10.1186/s12870-019-1756-4)

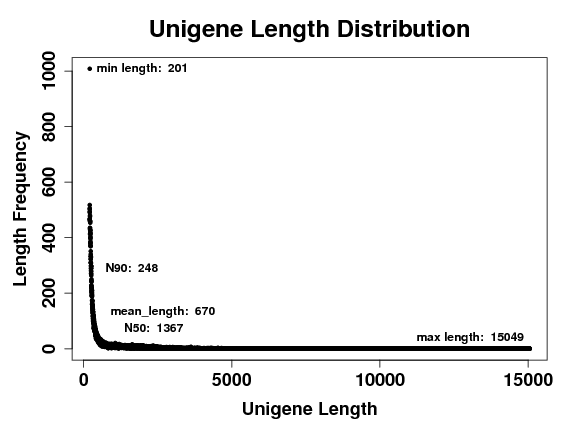


**Fig. S1.**Size distributions of unigenes in the reference library.

Supplement: Supplementary file 1 — Figure S1. Size distributions of unigenes in the reference library. (DOC 21 kb) [file 12870_2019_1756_MOESM1_ESM.doc]

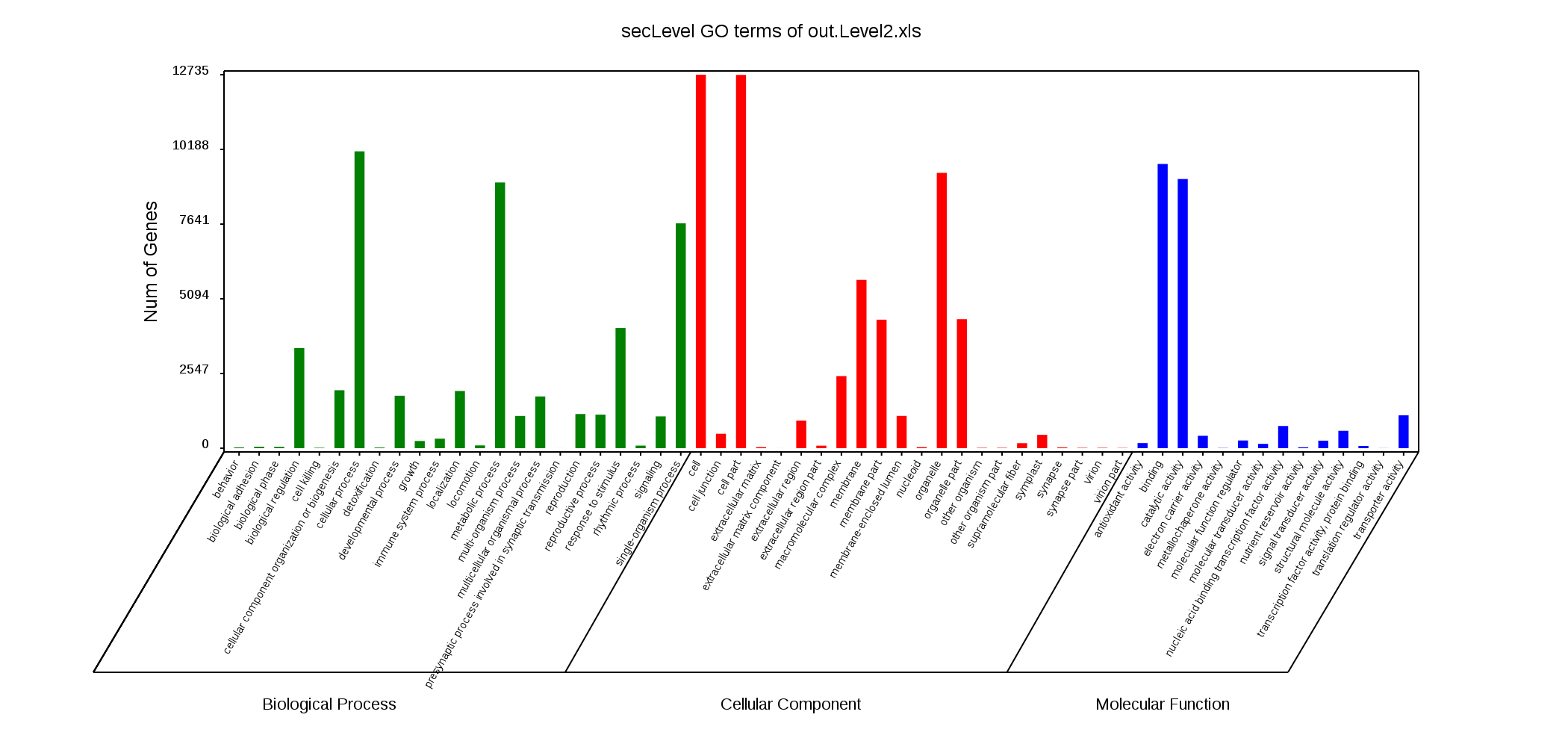


**Fig. S2.**GO assignment of all of the unigenes in the reference Library.

Supplement: Supplementary file 2 — Figure S2. GO assignment of all of the unigenes in the reference Library. (DOC 195 kb) [file 12870_2019_1756_MOESM2_ESM.doc]

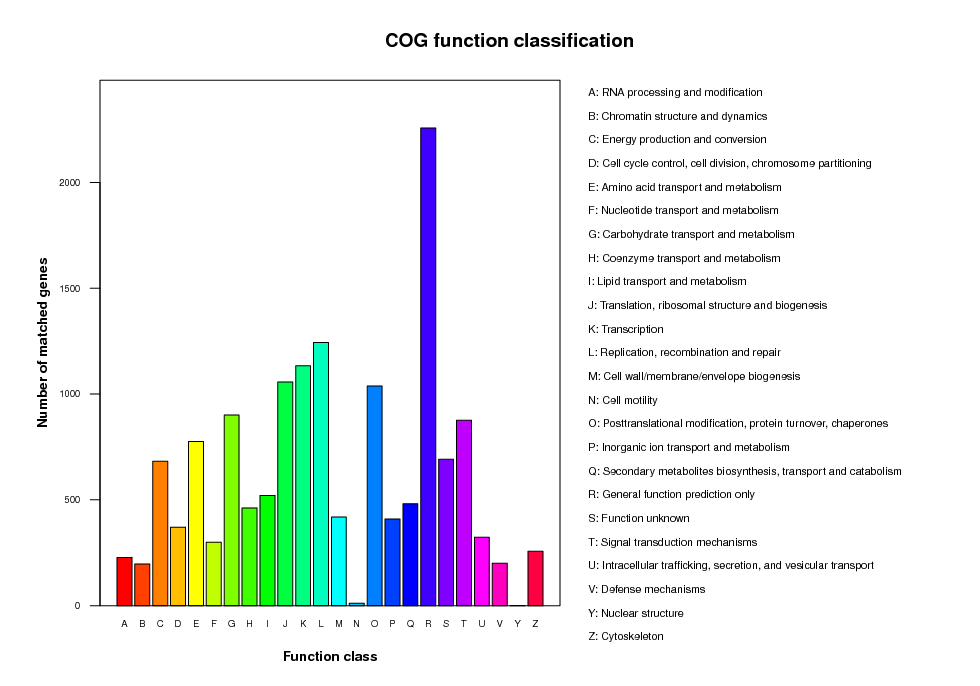


**Fig.S3.** COG assignment of all of the unigenes in the reference library.

Supplement: Supplementary file 3 — Figure S3. COG assignment of all of the unigenes in the reference library. (DOC 55 kb) [file 12870_2019_1756_MOESM3_ESM.doc]

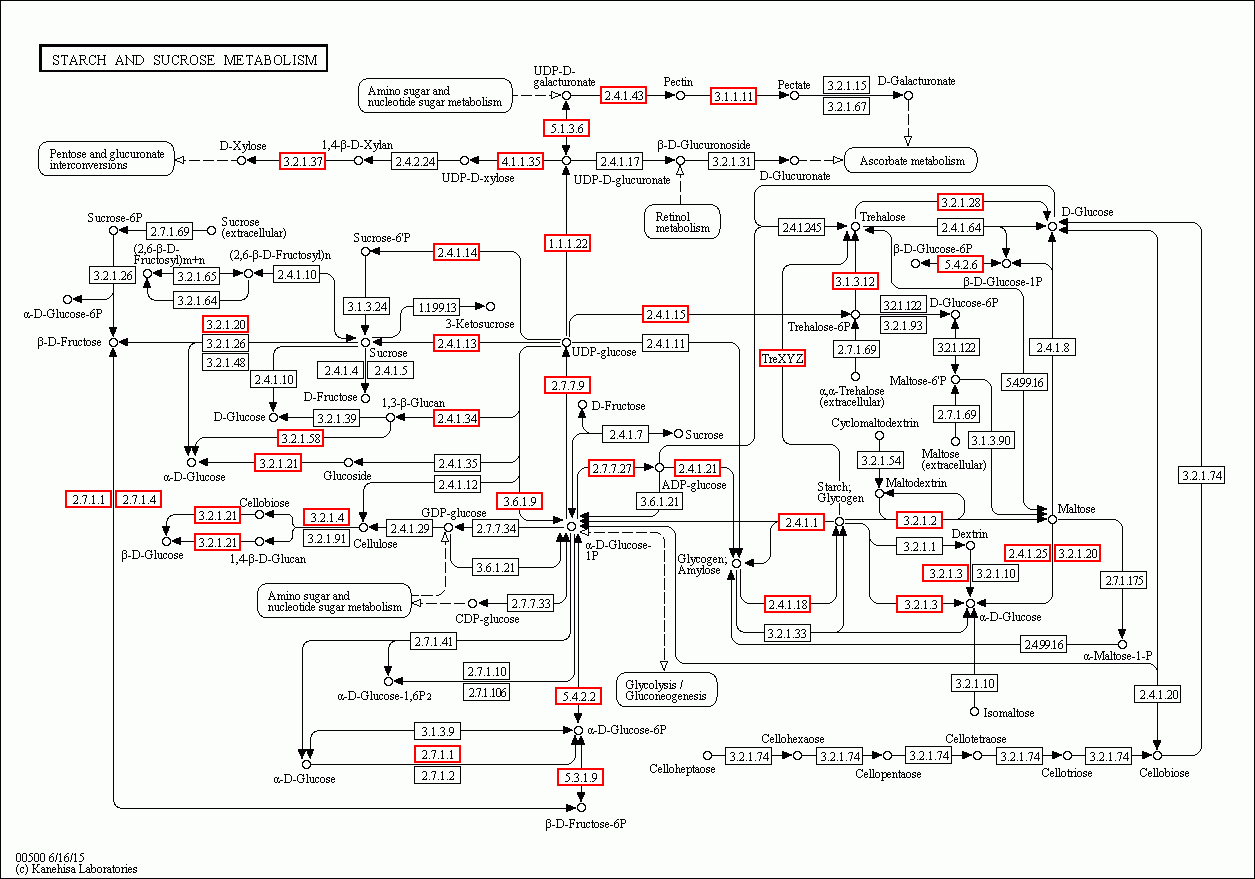


**Fig. S5.** Enriched starch and sucrose metabolism pathway.

Supplement: Supplementary file 8 — Figure S5. Enriched starch and sucrose metabolism pathway. The components marked with red rectangles were considered differentially expressed. (DOC 32 kb) [file 12870_2019_1756_MOESM8_ESM.doc]
