# Supplementary material for: Transcriptome analysis of atemoya pericarp elucidates the role of polysaccharide metabolism in fruit ripening and cracking after harvest
Source: BMC Plant Biol. 2019 May 27;19:219. doi: 10.1186/s12870-019-1756-4 (PMC6537181; doi:10.1186/s12870-019-1756-4)

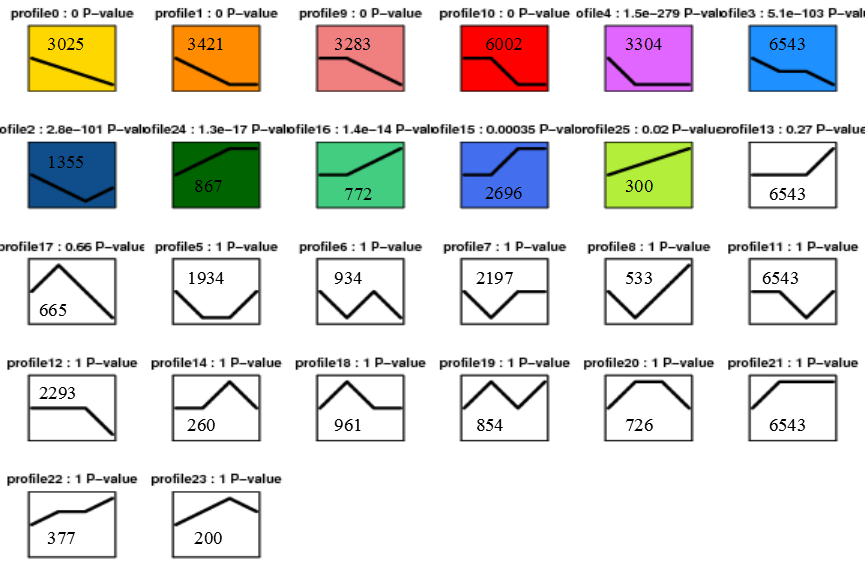


**Fig.S4.**Profiles ordered according to P-value significance of the number assigned versus expected.

**Additional file 8: Enriched starch and sucrose metabolism pathway. The signal transduction components marked with red rectangles are considered to be differentially expressed.**


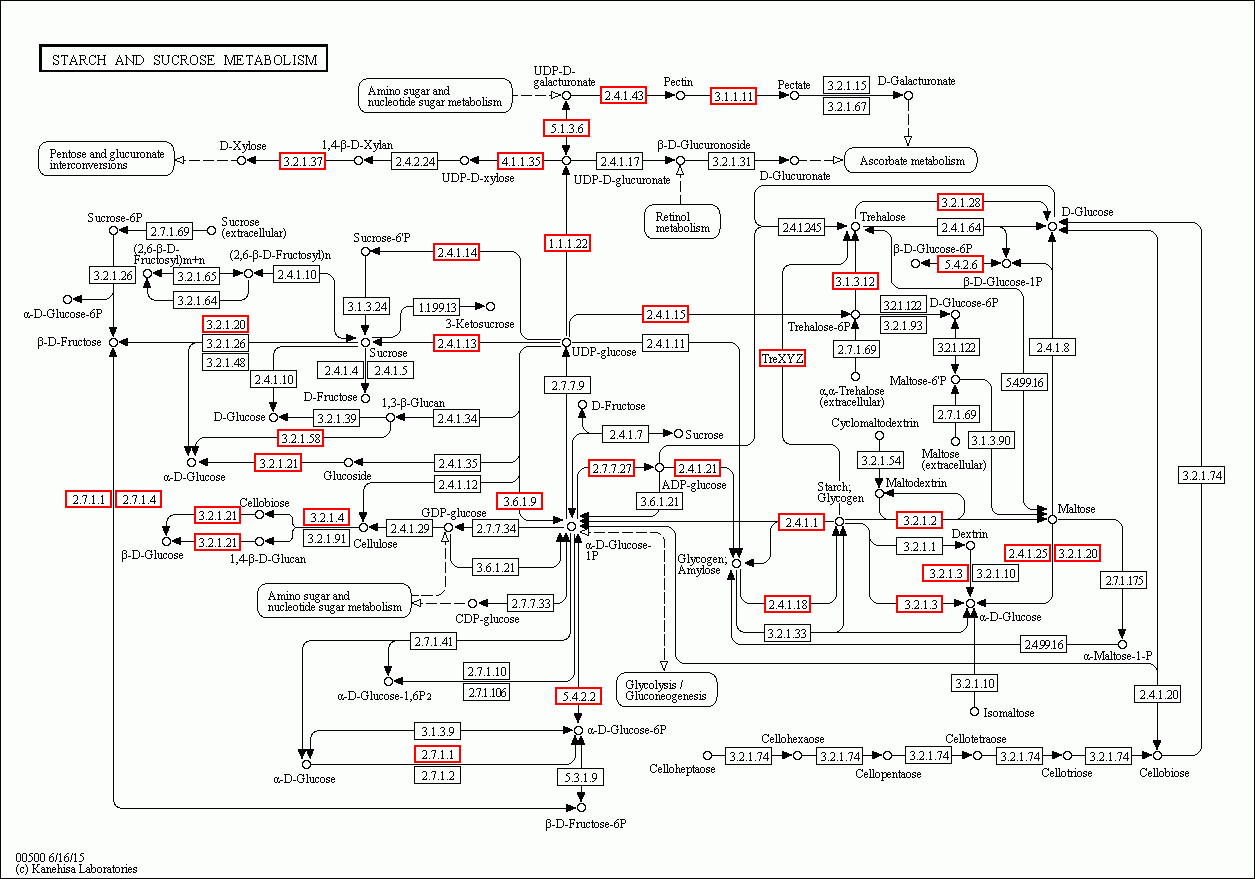

Supplement: Supplementary file 6 — Figure S4. Profiles ordered according to P-value significance of the number assigned versus expected. Numbers between brackets indicate the number of the DEGs assigned. (DOC 2023 kb) [file 12870_2019_1756_MOESM6_ESM.doc]
